# *TP53* gain-of-function mutations promote osimertinib resistance via TNF-α–NF-κB signaling in *EGFR*-mutated lung cancer

**DOI:** 10.1038/s41698-024-00557-2

**Published:** 2024-03-02

**Authors:** Ritsu Ibusuki, Eiji Iwama, Atsushi Shimauchi, Hirono Tsutsumi, Yasuto Yoneshima, Kentaro Tanaka, Isamu Okamoto

**Affiliations:** Department of Respiratory Medicine, Graduate School of Medical Sciences, Kyushu University, Fukuoka, Japan

**Keywords:** Non-small-cell lung cancer, Cancer therapeutic resistance

## Abstract

EGFR tyrosine kinase inhibitors (TKIs) are effective against *EGFR*-mutated lung cancer, but tumors eventually develop resistance to these drugs. Although *TP53* gain-of-function (GOF) mutations promote carcinogenesis, their effect on EGFR-TKI efficacy has remained unclear. We here established *EGFR*-mutated lung cancer cell lines that express wild-type (WT) or various mutant p53 proteins with CRISPR-Cas9 technology and found that *TP53*-GOF mutations promote early development of resistance to the EGFR-TKI osimertinib associated with sustained activation of ERK and expression of c-Myc. Gene expression analysis revealed that osimertinib activates TNF-α–NF-κB signaling specifically in *TP53*-GOF mutant cells. In such cells, osimertinib promoted interaction of p53 with the NF-κB subunit p65, translocation of the resulting complex to the nucleus and its binding to the *TNF* promoter, and TNF-α production. Concurrent treatment of *TP53*-GOF mutant cells with the TNF-α inhibitor infliximab suppressed acquisition of osimertinib resistance as well as restored osimertinib sensitivity in resistant cells in association with attenuation of ERK activation and c-Myc expression. Our findings indicate that induction of TNF-α expression by osimertinib in *TP53*-GOF mutant cells contributes to the early development of osimertinib resistance, and that TNF-α inhibition may therefore be an effective strategy to overcome such resistance in *EGFR*-mutant lung cancer with *TP53*-GOF mutations.

## Introduction

Lung cancer is the leading cause of cancer-related death worldwide, with non–small cell lung cancer (NSCLC) accounting for 80–85% of all lung cancer cases^1^. Activating mutations of the epidermal growth factor receptor gene (*EGFR*) have been detected in 30–40% of NSCLC tumors in Asian patients and in 10–15% of those in patients of European descent^2,3^. Osimertinib, a third-generation tyrosine kinase inhibitor (TKI) for EGFR, has shown greater efficacy compared with first-generation EGFR-TKIs and has become a standard treatment for *EGFR* mutation-positive NSCLC^4^. However, tumors inevitably develop resistance to osimertinib within 1–2 years. Although gene amplification of *MET* or *HER2* and transformation to small cell lung cancer have been identified in osimertinib-resistant tumors^5–8^, and cytokines such as tumor necrosis factor-α (TNF-α) and interferon have been associated with the development of EGFR-TKI resistance in NSCLC^9,10^, the mechanisms underlying most cases of osimertinib resistance have remained unclear.

*TP53* is a key tumor suppressor gene that contributes to regulation of the cell cycle, DNA repair, apoptosis, and senescence^11,12^. Mutation of *TP53* has been detected in 30–50% of individuals with *EGFR*-mutated lung cancer and has been associated with a shorter progression-free survival for EGFR-TKI treatment^13–16^. Most *TP53* mutations in cancer cells, including gain-of-function (GOF) mutations (such as R248Q, R273H, and R175H), result in single amino acid substitutions in the DNA binding domain of the encoded protein (p53)^17^. Such GOF mutations both result in the loss of tumor suppressor function of p53 and confer new functions that promote tumorigenesis, tumor invasiveness, and metastasis^18–23^.

To investigate whether GOF mutations of *TP53* contribute to attenuation of the therapeutic efficacy of osimertinib, we have now examined the sensitivity to and survival during osimertinib treatment for *EGFR*-mutated lung cancer cell lines engineered to harbor various *TP53* mutations, including GOF and non-GOF mutations. We also performed comprehensive investigations including RNA-sequencing (seq) and chromatin immunoprecipitation (ChIP)-seq analyses to probe the mechanism by which such mutations were found to confer osimertinib resistance.

## Results

### Establishment of p53-KO and p53-mutant cell lines

**Fig. 1:**
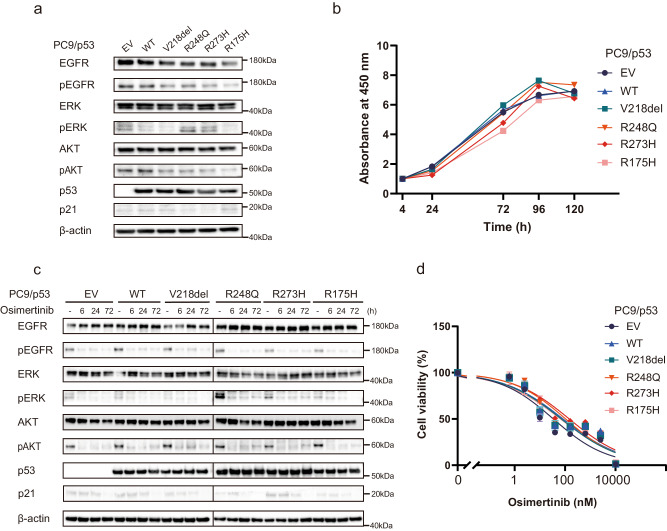
*TP53* status does not affect osimertinib sensitivity of *EGFR*-mutated NSCLC cells. **a** Immunoblot analysis of p53, p21, and total and phosphorylated (p) forms of EGFR, ERK, and AKT in PC9/p53^EV^, PC9/p53^WT^, and PC9/p53^MUT^ cells. β-actin was examined as a loading control. **b** Cell proliferation curves for PC9/p53^EV^, PC9/p53^WT^, and PC9/p53^MUT^ cells determined with a colorimetric assay. **c** Immunoblot analysis of EGFR signaling as well as of p53 and p21 in PC9/p53^EV^, PC9/p53^WT^, and PC9/p53^MUT^ cells treated with 100 nM osimertinib for 0, 6, 24, or 72 h. **d** Viability of PC9/p53^EV^, PC9/p53^WT^, and PC9/p53^MUT^ cells exposed to the indicated concentrations of osimertinib for 72 h as determined with a colorimetric assay. Data in (**b**) and (**d**) are means ± SEM of triplicates from one experiment and are representative of three independent experiments.

We first examined the frequency and type of *TP53* mutations in patients with advanced NSCLC positive for activating mutations (L858R or exon-19 deletions) of *EGFR* with the use of data from the MSK-MET study^24^ that were accessed via the cBioPortal database^25,26^. Among 876 patients with *EGFR* activating mutations, 504 (57.5%) individuals were found to harbor *TP53* mutations, with missense mutations at hot spot locations (R273, R248, and R175) being prominent (Supplementary Fig. 1a). These missense mutations included the well-established *TP53*-GOF mutations R273H, R248Q, and R175H^27,28^. We next deleted the endogenous *TP53* gene in the *EGFR*-mutant human NSCLC cell line PC-9 using CRISPR-Cas9 technology. The resulting PC9/p53^KO^ cells were then infected with retroviruses encoding wild-type (WT) or various mutant (V218del, R248Q, R273H, or R175H) forms of human p53, or with the empty vector (EV), to yield PC9/p53^WT^, PC9/p53^MUT^, or PC9/p53^EV^ cell lines, respectively (Fig. 1a). We also established corresponding cell lines that express p53 in a doxycycline-inducible manner (PC9/tetO-p53^WT^, PC9/tetO-p53^MUT^, and PC9/tetO-p53^EV^, respectively) (Supplementary Fig. 1b). The expression level of p53 induced by doxycycline or in nonengineered cell lines was greater for cells harboring *TP53*-GOF mutations (R248Q, R273H, or R175H) than for those WT for or harboring non-GOF mutations of *TP53* (Supplementary Fig. 1b, c), consistent with previous findings^29^. Whereas p21, the product of a major target gene of p53, was highly expressed in the *TP53-*WT cell line A549 and the *TP53*-Y205H cell line HCC4006 (Supplementary Table 1), and it was highly induced by doxycycline in PC9/tetO-p53^WT^ cells, its expression was low in most *TP53*-mutant cell lines and was not induced in PC9/tetO-p53^MUT^ cell lines, indicating that the ability of p53 to activate p21 expression was lost in these mutant cells (Supplementary Fig. 1b, c).

### *TP53* status does not affect intrinsic osimertinib sensitivity of *EGFR*-mutant NSCLC cells

Although slight differences in EGFR signaling (as reflected by the phosphorylation of EGFR, ERK, and AKT) at baseline were observed according to *TP53* status (Fig. 1a), no substantial differences in cell proliferation rate were apparent among the PC9 cell lines forcibly expressing p53 constitutively (Fig. 1b). Similar results were obtained for the PC9 cell lines expressing p53 inducibly (Supplementary Figs. 1b and 2a). Osimertinib treatment effectively inhibited EGFR, ERK, and AKT phosphorylation irrespective of *TP53* status in the PC9 cells constitutively expressing p53 (Fig. 1c). The concentration-dependent inhibition of cell viability apparent after exposure to osimertinib for 72 h did not differ according to *TP53* status in PC9 cells constitutively or inducibly expressing p53 (Fig. 1d and Supplementary Fig. 2b). In addition, no differences in sensitivity to osimertinib were observed according to *TP53* genotype in other *EGFR*-mutated NSCLC cell lines (HCC4006 and H1975) stably expressing various p53 mutant proteins (Supplementary Figs. 1d, e and 2c, d). These findings suggested that *TP53* status did not affect the survival of *EGFR*-mutated NSCLC cells exposed to osimertinib for up to 72 h.

### *TP53*-GOF mutations confer early acquisition of resistance to osimertinib

**Fig. 2:**
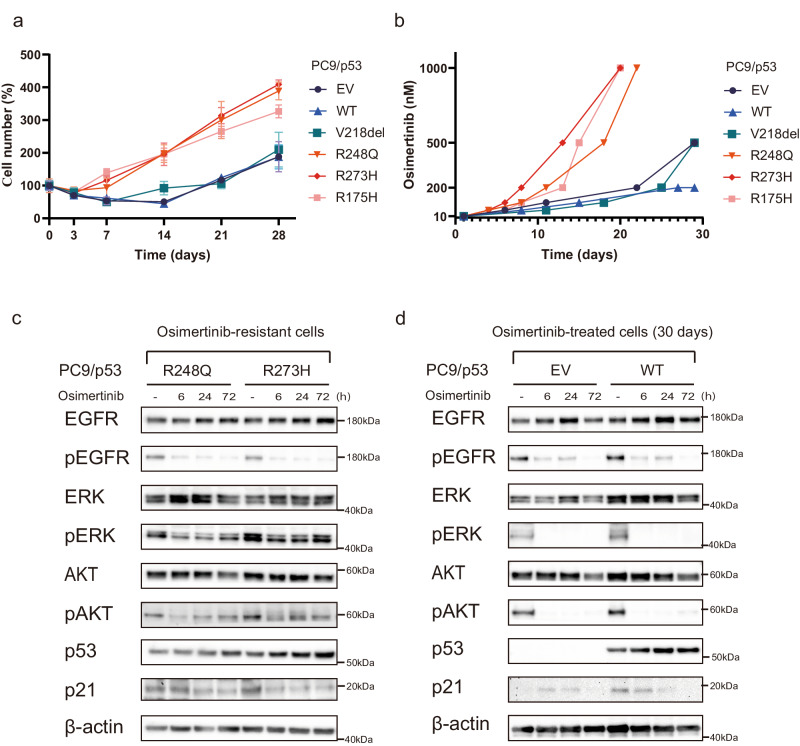
*TP53*-GOF mutation promotes the early development of osimertinib resistance in *EGFR*-mutant NSCLC cells. **a** Time course of PC9/p53^EV^, PC9/p53^WT^, and PC9/p53^MUT^ cell number during treatment with 1 μM osimertinib for up to 28 days. **b** Time course for the development of osimertinib resistance in PC9/p53^EV^, PC9/p53^WT^, and PC9/p53^MUT^ cells. The concentration of osimertinib was gradually increased from 10 nM to 1 μM each time the cells achieved 70% confluence. Immunoblot analysis of EGFR signaling as well as of p53 and p21 during treatment with 1 μM osimertinib for up to 72 h for osimertinib-resistant PC9/p53^GOF^ cells (**c**) and PC9/p53^EV^ and PC9/p53^WT^ cells that had been previously treated with osimertinib for 30 days (**d**). Data in (**a**) and (**b**) are means ± SEM for triplicates from one experiment and are representative of two independent experiments.

Given the possibility that longer exposure of cells to osimertinib might reveal an effect of *TP53* status on drug action, we treated PC9/p53 cell lines with 1 µM osimertinib for up to 28 days. Whereas osimertinib effectively suppressed cell proliferation irrespective of *TP53* status during treatment for up to 3 days, the proliferation of PC9/p53^GOF^ cells recovered faster and increased to a greater extent compared with that of PC9/p53^EV^, PC9/p53^V218del^, or PC9/p53^WT^ cells during continuous exposure to the drug (Fig. 2a). Similar results were obtained with both HCC4006 and H1975 cell lines stably expressing various types of p53 protein (Supplementary Fig. 3). We next subjected the PC9 cell lines to gradual escalation of osimertinib concentration, with the concentration being increased in stages from 10 nM up to 1 µM each time the cells achieved 70% confluence. This protocol revealed that PC9/p53^GOF^ cells developed resistance to 1 µM osimertinib substantially earlier, at ~20 days after treatment initiation, than did the other cell lines (Fig. 2b). The development of resistance to 1 µM osimertinib was not observed after 30 days of treatment with the drug in PC9/p53^EV^ or PC9/p53^WT^ cells. EGFR downstream signaling including the phosphorylation of ERK was attenuated but relatively sustained in the osimertinib-resistant PC9/p53^GOF^ cell lines, compared with PC9/p53^EV^ and PC9/p53^WT^ cells treated with osimertinib for 30 days, during subsequent exposure to 1 µM osimertinib for up to 72 h (Fig. 2c, d). The expression level of HER2 or MET did not differ among PC9/p53^EV^, PC9/p53^WT^, and PC9/p53^MUT^ cells at baseline (Supplementary Fig. 4a), and expression of these proteins was not increased in PC9/p53^R248Q^ and PC9/p53^R273H^ cells after the acquisition of resistance to osimertinib (Supplementary Fig. 4b). These findings suggested that amplification of *HER2* or *MET* was not responsible for the development of resistance to osimertinib in PC9/p53^GOF^ cells.

### *TP53*-GOF mutation activates TNF-α–NF-κB signaling during osimertinib treatment

**Fig. 3:**
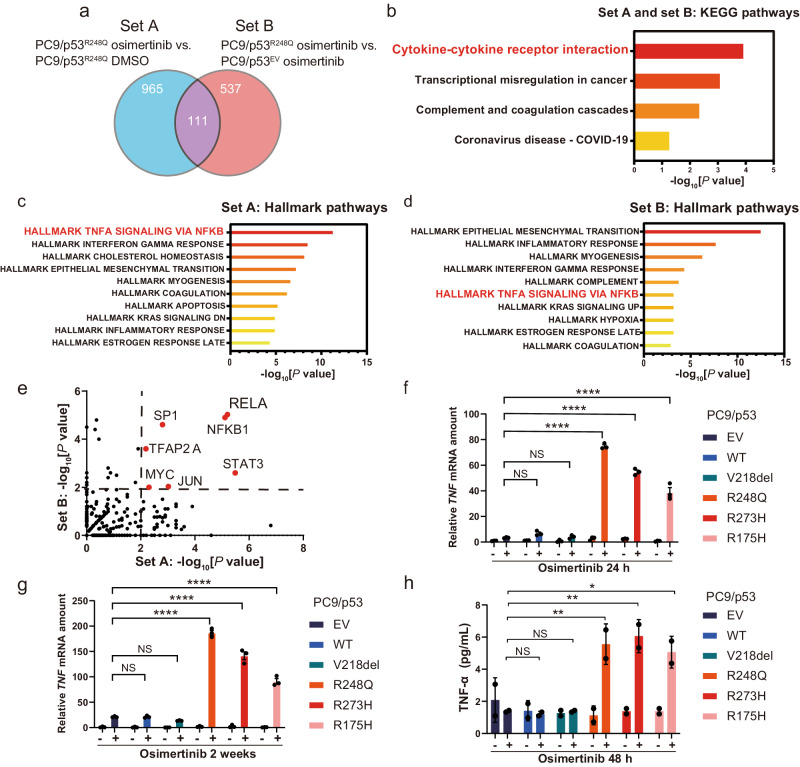
*TP53*-GOF mutation promotes activation of TNF-α–NF-κB signaling by osimertinib in *EGFR*-mutated NSCLC cells. **a** Venn diagram showing the overlap in the number of significantly upregulated (log_2_[fold change] > 1, *p* < 0.05) genes in set A (PC9/p53^R248Q^ cells incubated with 600 nM osimertinib for 24 h versus those incubated with DMSO vehicle) and in set B (PC9/p53^R248Q^ cells versus PC9/p53^EV^ cells, each exposed to 600 nM osimertinib for 24 h) as determined by RNA-seq. **b** KEGG pathway analysis for the 111 genes commonly upregulated in sets A and B. Hallmark pathways with the highest −log_10_[*p* values] are shown. Pathway enrichment analysis for the significantly upregulated genes in set A (**c**) and set B (**d**). Hallmark gene sets with the highest −log_10_[*p* values] are shown. **e** Volcano plot based on −log_10_[*p* value] for TRRUST analysis of set A and set B. The dashed lines indicate a −log_10_[*p* value] of 2 (*p* = 0.01). RT-qPCR analysis of *TNF* mRNA abundance in PC9/p53^EV^, PC9/p53^WT^, and PC9/p53^MUT^ cells incubated with or without 600 nM osimertinib for 24 h (**f**) or for 2 weeks (**g**). Data are means ± SEM of triplicates from one experiment and are representative of three independent experiments. **h** Concentration of TNF-α in serum-free culture supernatants of PC9/p53^EV^, PC9/p53^WT^, and PC9/p53^MUT^ cells incubated in the absence or presence of 600 nM osimertinib for 48 h. Data are means ± SD for duplicates from one experiment and are representative of two independent experiments. *p < 0.05, **p < 0.01, ****p < 0.0001, NS not significant (one-way ANOVA followed by Tukey’s test).

To investigate the mechanism by which *TP53*-GOF mutation promotes the development of osimertinib resistance, we performed RNA-seq analysis for examination of the upregulation of gene expression in PC9/p53^R248Q^ cells incubated with 600 nM osimertinib versus those incubated with dimethyl sulfoxide (DMSO) vehicle for 24 h (set A) as well as in osimertinib-treated PC9/p53^R248Q^ cells versus osimertinib-treated PC9/p53^EV^ cells (set B). The expression of a total of 111 genes (Supplementary Table 2) was commonly upregulated in both sets A and B (Fig. 3a). Kyoto Encyclopedia of Genes and Genomes (KEGG) pathway enrichment analysis revealed that cytokine–cytokine receptor interaction was the most significantly enriched pathway among these 111 genes (Fig. 3b). Hallmark pathway analysis identified the TNF-α–NF-κB signaling pathway as being significantly enriched in set A (Fig. 3c) and in set B (Fig. 3d). Furthermore, TRRUST analysis of both gene sets indicated increased activity of transcription factors including the NF-κB subunits RELA (p65) and NFKB1 (Fig. 3e). Reverse transcription and quantitative polymerase chain reaction (RT-qPCR) analysis of gene expression for cytokines related to tumor progression confirmed that *TNF* mRNA abundance was increased specifically in PC9/p53^GOF^ cells treated with osimertinib (Supplementary Fig. 5). Although the upregulation of *TNF* expression was apparent 24 h after the onset of exposure to 600 nM osimertinib in PC9/p53^GOF^ (Fig. 3f) and PC9/tetO-p53^GOF^ (Supplementary Fig. 6a) cell lines, the extent of this upregulation was much greater (up to ~200-fold increase) after continuous exposure of the cells to osimertinib for 2 weeks (Fig. 3g). Enzyme-linked immunosorbent assay (ELISA) analysis also revealed that the amount of TNF-α was specifically increased in culture supernatants of PC9/p53^GOF^ cell lines after osimertinib treatment for 48 h (Fig. 3h). The upregulation of *TNF* expression after exposure to osimertinib was also specifically observed in both HCC4006 and H1975 cells stably expressing p53^GOF^ mutants (Supplementary Fig. 6b, c). The p65 inhibitor JSH-23 suppressed the increase in *TNF* mRNA abundance induced by osimertinib treatment in PC9/p53^R248Q^ cells (Supplementary Fig. 6h). Together, these findings thus suggested that *TP53*-GOF mutation promotes the activation of TNF-α–NF-κB signaling in cells subjected to continuous osimertinib treatment.

### Osimertinib promotes binding of p53 and p65 to the *TNF* promoter in *TP53*-GOF mutant cells

**Fig. 4:**
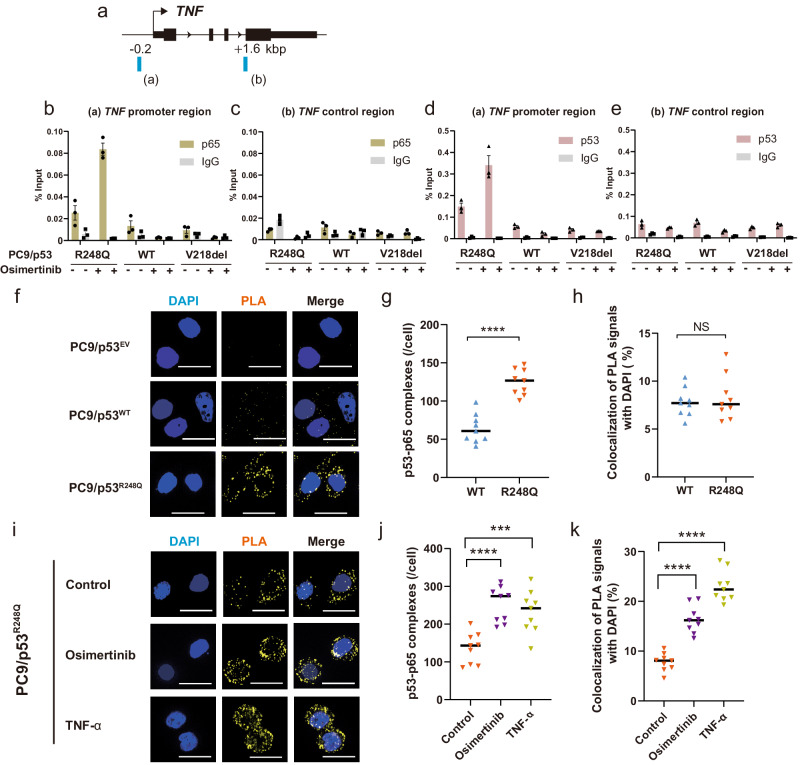
Osimertinib upregulates *TNF* expression in *TP53*-GOF mutant cells by inducing p53-p65 interaction. **a** Schematic representation of ChIP-qPCR analysis. ChIP-qPCR primers were designed to amplify promoter (**a**) or control (**b**) regions of the *TNF* gene locus. Percentage of ChIP-qPCR amplicons derived from the promoter (**b**, **d**) or control (**c**, **e**) regions of *TNF* that were immunoprecipitated with antibodies to p65 (**b**, **c**) or to p53 (**d**, **e**), or with control IgG, from PC9/p53^WT^, PC9/p53^V218del^, or PC9/p53^R248Q^ cells incubated with or without 600 nM osimertinib for 24 h. Data are means ± SEM of triplicates from one experiment and are representative of two independent experiments. Fluorescence microscopic images of p53-p65 complexes (yellow) detected by an in situ PLA for PC9/p53^EV^, PC9/p53^WT^, and PC9/p53^R248Q^ cells under basal conditions (**f**) or for PC9/p53^R248Q^ cells incubated in the absence or presence of osimertinib (600 nM) or TNF-α (1 ng/ml) for 24 h (**i**). Nuclei were stained with DAPI (blue). The representative images were obtained by optical sectioning. Scale bars, 20 μm. Number of p53-p65 complexes per cell (**g**, **j**) and percentage colocalization of PLA signals with DAPI staining (**h**, **k**) determined from *z*-projection images constructed by *z*-stacking of optical sections for cells as in (**f**) and (**i**), respectively. Data are means ± SEM (*n* = 9 fields including a total of at least 50 cells). ***p < 0.001, ****p < 0.0001, NS (one-way ANOVA followed by Tukey’s test).

Given that TRRUST analysis implicated RELA (p65) as a key transcription factor influenced by *TP53*-GOF mutation and osimertinib treatment in *EGFR*-mutated NSCLC cells, we examined the binding of p65 to the promoter region of the *TNF* gene by ChIP-qPCR analysis. The p65 binding motif was identified at a position of about −0.2 kbp (relative to the transcription start site) in the promoter region of *TNF* by JASPAR (http://jaspar.genereg.net)^30^, and a negative control sequence was set at a position of +1.6 kbp (Fig. 4a). We detected specific binding of p65 to the *TNF* promoter region in PC9/p53^R248Q^ cells treated with osimertinib (Fig. 4b, c). Given that p53 and p65 have previously been shown to bind to each other^31–33^, we also examined the possible interaction of p53 with the *TNF* promoter region. We found that p53 indeed also bound specifically to this region in the *TP53*-GOF mutant cells treated with osimertinib (Fig. 4d, e). Similar results were obtained with PC9/p53^R273H^ and PC9/p53^R175H^ cells treated with osimertinib (Supplementary Fig. 6d–g). These findings thus indicated that osimertinib treatment promoted the binding of both p53 and p65 to the promoter region of the *TNF* gene in *TP53*-GOF mutant cells.

### Osimertinib and TNF-α promote interaction of p53 and p65 in *TP53*-GOF mutant cells

We next performed a proximity ligation assay (PLA) to detect the interaction of p53 and p65. The number of p53-p65 complexes per cell was found to be significantly greater in PC9/p53^R248Q^ cells compared with PC9/p53^WT^ cells (Fig. 4f, g). The percentage of PLA signals for these complexes that colocalized with nuclear staining with 4′,6-diamidino-2-phenylindole (DAPI) did not differ between PC9/p53^R248Q^ and PC9/p53^WT^ cells (Fig. 4h), however, indicating that *TP53*-GOF mutation did not induce translocation of p53-p65 complexes to the nucleus in the absence of osimertinib treatment. On the other hand, treatment with osimertinib (600 nM) or TNF-α (1 ng/ml) for 24 h increased the number of p53-p65 complexes per cell and promoted translocation of these complexes to the nucleus in PC9/p53^R248Q^ cells (Fig. 4i–k). Collectively, these findings suggested that translocation of p65 to the nucleus induced by osimertinib promotes *TNF* expression in PC9/p53^R248Q^ cells via formation of a p53-p65 complex.

### Osimertinib treatment promotes binding of p53^R248Q^ to the p65 binding motif

**Fig. 5:**
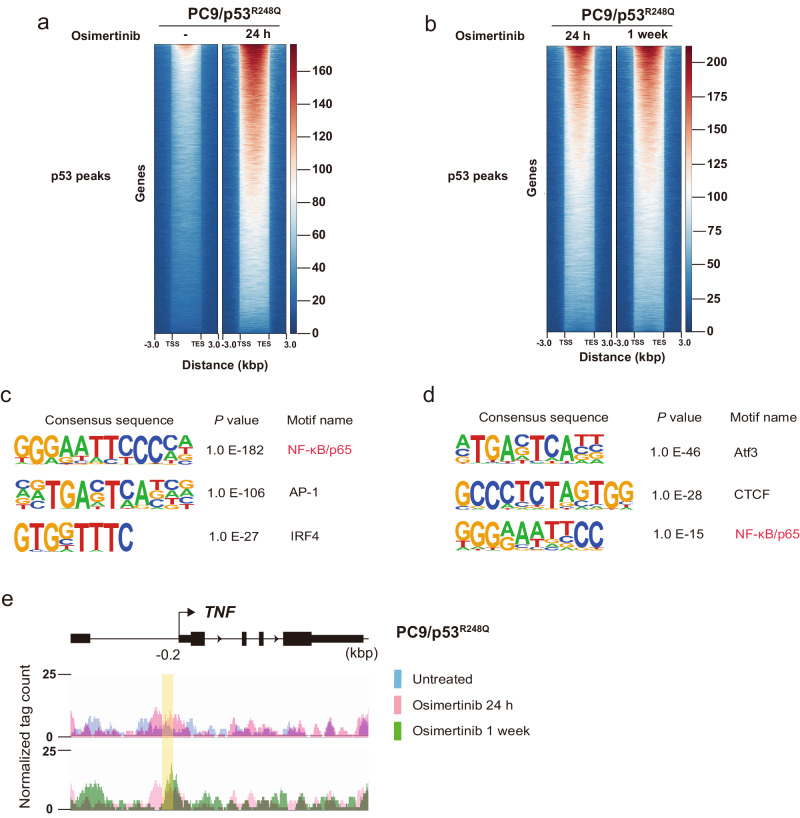
Osimertinib induces the binding of p53 to p65 binding sites in DNA of PC9/p53^R248Q^ cells. Aggregation maps of p53 ChIP-seq reads for PC9/p53^R248Q^ cells treated with osimertinib for 0 or 24 h (**a**) or for 24 h or 1 week (**b**). Each row shows a region of ±3 kbp centered on a p53 peak, with the rows being rank-ordered on the basis of peak intensity. TSS transcription start site, TES transcription end site. De novo motif analysis for p53 binding peaks enriched in PC9/p53^R248Q^ cells treated with osimertinib for 24 h versus those treated for 0 h (**c**) as well as in those treated with osimertinib for 1 week versus those treated for 24 h (**d**). The top three consensus sequences are shown in rank order by *p* value. **e** IGV genome browser tracks of ChIP-seq signals for p53 at the *TNF* gene locus in PC9/p53^R248Q^ cells treated with osimertinib for 0 h (blue), 24 h (pink), or 1 week (green). The *y*-axis depicts the ChIP-seq signal, and the *x*-axis the genomic position. The position of the p65 binding motif detected by JASPAR in the promoter region of *TNF* (−0.2 kbp) as shown in Fig. 4a is highlighted in yellow.

To investigate further the interaction of p53-GOF mutant proteins with the genome induced by osimertinib treatment, we performed ChIP-seq analysis for PC9/p53^R248Q^ cells treated (or not) with osimertinib (600 nM) for 24 h or 1 week. Osimertinib treatment for 24 h markedly increased the binding of p53 to various genes in PC9/p53^R248Q^ cells (Fig. 5a), with this effect being more pronounced after exposure of the cells to osimertinib for 1 week (Fig. 5b). De novo motif analysis revealed that the NF-κB/p65 consensus motif was highly enriched among the p53 peaks in PC9/p53^R248Q^ cells treated with osimertinib for 24 h compared with untreated cells (Fig. 5c) as well as among those in the cells treated with osimertinib for 1 week relative to the cells treated for 24 h (Fig. 5d). Integrated Genomics Viewer (IGV) genome browser tracks of ChIP-seq signals showed the binding of p53 to the promoter region of *TNF* including the p65 binding site (−0.2 kbp) detected by JASPAR (Fig. 4a), with such binding to this site being increased by osimertinib treatment in a time-dependent manner (Fig. 5e). These findings indicated that osimertinib treatment induces the binding of p53 to the p65 binding site of the *TNF* promoter region by promoting its interaction with p65.

### Inhibition of TNF-α suppresses the development of resistance and restores sensitivity to osimertinib in *TP53*-GOF mutant cells

**Fig. 6:**
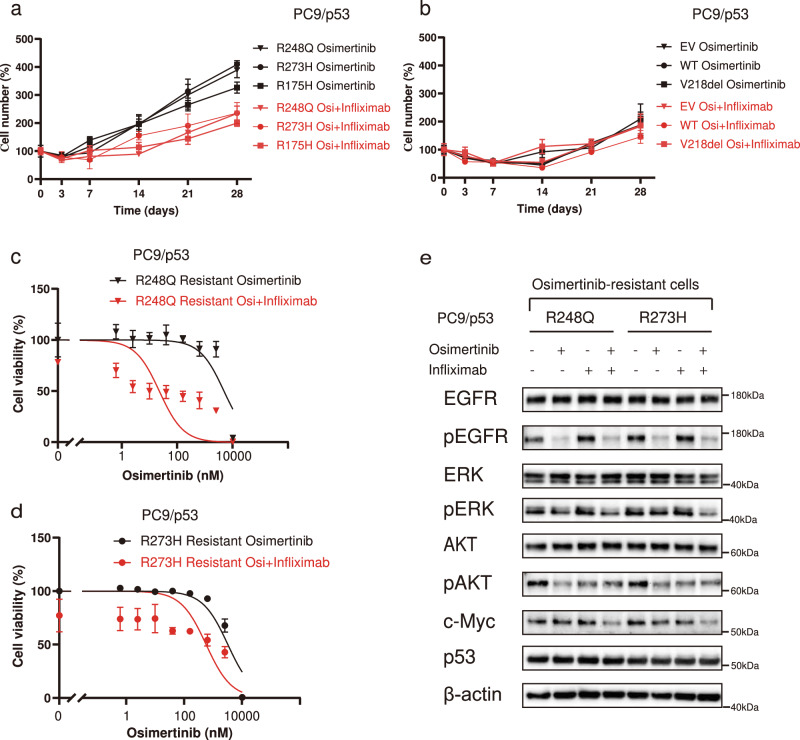
Inhibition of TNF-α signaling suppresses the development of resistance and restores sensitivity to osimertinib in *TP53*-GOF mutant cells. Percentage cell number for PC9/p53^GOF^ mutant cells (**a**) as well as PC9/p53^EV^, PC9/p53^WT^, and PC9/p53^V218del^ cells (**b**) treated with 1 μM osimertinib (Osi) in the absence or presence of infliximab (1 µg/ml) for the indicated times. Viability of osimertinib-resistant PC9/p53^R248Q^ (**c**) and PC9/p53^R273H^ (**d**) cells treated with the indicated concentrations of osimertinib in the absence or presence of infliximab (1 µg/ml) for 72 h as measured by a colorimetric assay. **e** Immunoblot analysis of EGFR signaling as well as p53 and c-Myc expression in osimertinib-resistant PC9/p53^GOF^ mutant cells incubated in the absence or presence of osimertinib (100 nM) or infliximab (1 µg/ml) for 24 h. Data in (**a**) through (**d**) are means ± SEM of triplicates from one experiment and are representative of three independent experiments.

Given that upregulation of TNF-α expression by osimertinib treatment was specific to *TP53*-GOF mutant cells, we examined whether TNF-α might contribute to the early development of resistance to osimertinib in such cells by determining the effects of inhibition of TNF-α. We first investigated the effect of combination treatment with osimertinib and the anti-TNF-α antibody infliximab on cell viability in PC9/p53 cells. Infliximab treatment alone did not show cytotoxicity over 72 h (Supplementary Fig. 7a) or a long-term inhibitory effect on cell growth (Supplementary Fig. 7b). In addition, the presence of infliximab did not affect the sensitivity of PC9/p53^EV^ or PC9/p53^R248Q^ cell lines to osimertinib during incubation for 72 h (Supplementary Fig. 7c, d). In contrast, the development of osimertinib resistance induced by long-term drug exposure in PC9/p53^GOF^ cell lines was suppressed in the presence of infliximab (Fig. 6a), whereas such effects were not observed in PC9/p53^EV^, PC9/p53^WT^, or PC9/p53^V218del^ cells (Fig. 6b). Furthermore, combination treatment with infliximab restored sensitivity to osimertinib in osimertinib-resistant PC9/p53^GOF^ mutant cells (Fig. 6c, d). Whereas treatment with osimertinib alone did not suppress ERK phosphorylation and c-Myc expression in the osimertinib-resistant cell lines, the combination treatment effectively inhibited such signaling (Fig. 6e). Treatment with TNF-α promoted the development of osimertinib resistance in parental PC-9 (*TP53*-GOF) and HCC4006 (*TP53* non-GOF) cells (Supplementary Fig. 8). These results indicated that TNF-α contributes to the development of osimertinib resistance in cells with *EGFR* mutations, with TNF-α production being increased by exposure to osimertinib in such cells with coexisting *TP53*-GOF mutations.

### Infliximab suppresses the development of resistance and restores sensitivity to osimertinib in a xenograft mouse model

**Fig. 7:**
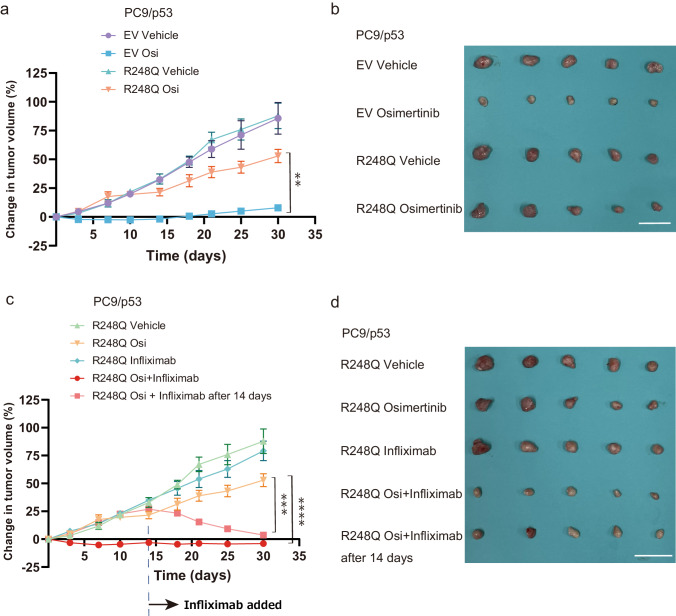
Inhibition of TNF-α signaling suppresses the development of resistance and restores sensitivity to osimertinib in a xenograft mouse model. **a** Time course of changes in tumor volume for subcutaneous tumors formed by PC9/p53^EV^ or PC9/p53^R248Q^ cells in nude mice and treated with vehicle or osimertinib (Osi) beginning at day 0 (corresponding to 10 days after cell injection). **b** Tumors isolated from mice in (**a**) at day 30. Scale bar, 30 mm. **c** Time course of changes in tumor volume for subcutaneous tumors formed by PC9/p53^R248Q^ cells in nude mice and treated with vehicle, osimertinib, infliximab, or the combination of osimertinib and infliximab from day 0. Alternatively, infliximab administration was initiated 14 days after the onset of osimertinib treatment. **d** Tumors isolated from mice in (**c**) at day 30. Scale bar, 30 mm. For these experiments, osimertinib was administered orally once daily at a dose of 1 mg/kg and infliximab was administered intraperitoneally once a week at a dose of 10 mg/kg. All quantitative data are means ± SEM (*n* = 5 mice per group). **p < 0.01, ***p < 0.001, ****p < 0.0001 (one-way ANOVA followed by Tukey’s test).

Finally, we performed in vivo experiments with a xenograft mouse model based on subcutaneous injection of PC9/p53 cells into athymic nude mice. Whereas the growth of tumors formed by PC9/p53^EV^ cells was effectively suppressed by treatment with osimertinib, tumors formed by PC9/p53^R248Q^ cells rapidly acquired osimertinib resistance (Fig. 7a, b). We confirmed that tumors formed by PC9/p53^R248Q^ cells in this mouse model showed a significant increase in *TNF* expression after treatment with osimertinib for 2 weeks as compared with tumors formed by PC9/p53^EV^ cells (Supplementary Fig. 9a). The rapid development of osimertinib resistance by PC9/p53^R248Q^ tumors was effectively inhibited by simultaneous treatment with infliximab (Fig. 7c, d). Of note, treatment with infliximab even restored the sensitivity of PC9/p53^R248Q^ tumors to osimertinib after the development of osimertinib resistance (Fig. 7c, d). Treatment with the combination of osimertinib and infliximab appeared to be well tolerated, with no mice experiencing weight loss of ≥10% (Supplementary Fig. 9b, c).

## Discussion

**Fig. 8:**
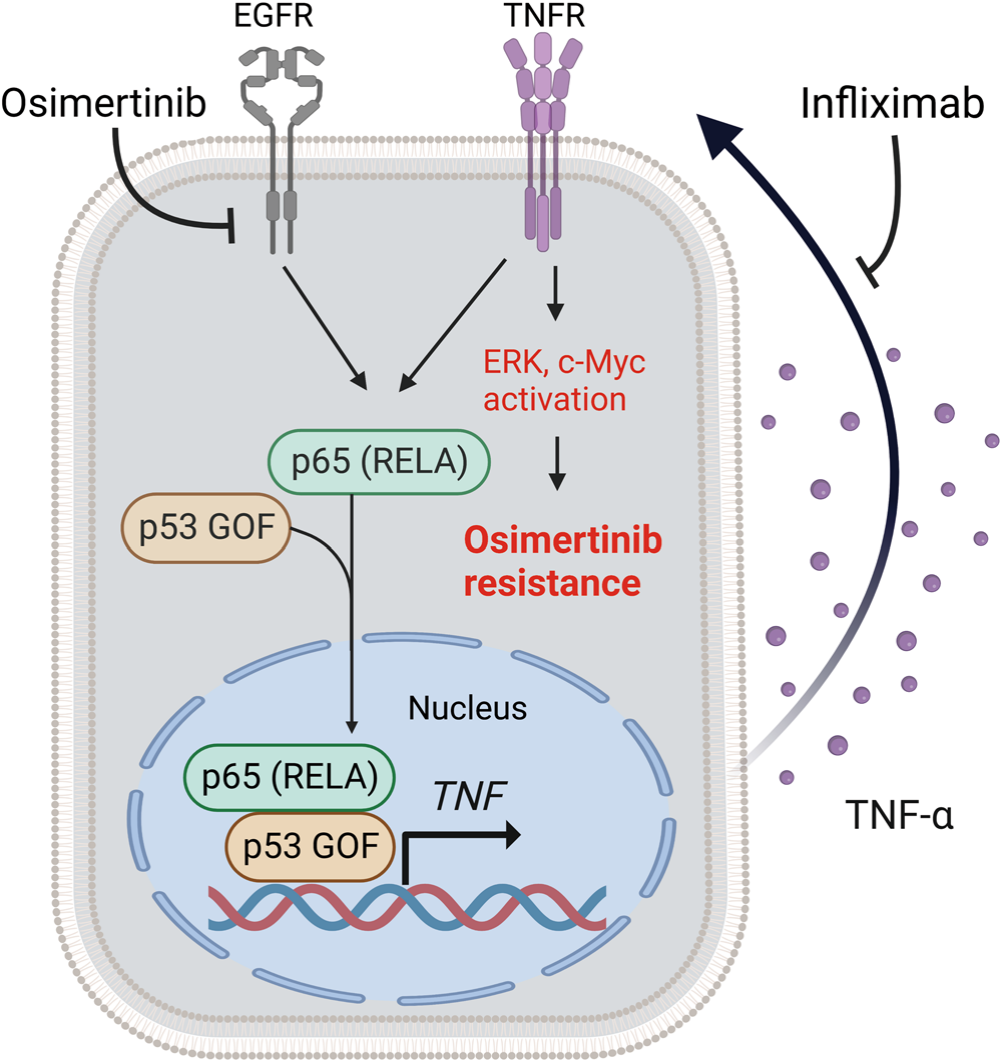
Mechanism of osimertinib resistance in *EGFR*-mutated lung cancer with coexisting *TP53*-GOF mutations. Osimertinib treatment induces p65 activation, whereas *TP53*-GOF mutations promote formation of a p53-p65 complex that binds to the *TNF* promoter region and thereby increases TNF-α production in osimertinib-treated cells. TNF-α then not only promotes formation and nuclear translocation of the p53-p65 complex but also activates ERK and c-Myc, giving rise to osimertinib resistance. The anti-TNF-α antibody infliximab inhibits TNF-α signaling and thereby suppresses osimertinib resistance.

We have here shown that *TP53*-GOF mutations promote the formation and nuclear translocation of a p53-p65 complex in response to osimertinib treatment. This complex binds to the promoter region of the *TNF* gene, and thereby likely mediates the induction of TNF-α expression by osimertinib in *TP53*-GOF mutant cells. TNF-α was also found to promote formation of the p53-p65 complex and its translocation to the nucleus, suggesting that osimertinib induces TNF-α production through a positive feedback mechanism mediated by the TNF-α–NF-κB signaling pathway in NSCLC cells with *TP53*-GOF mutations (Fig. 8). We also found that *TP53*-GOF mutations accelerate the development of resistance to osimertinib and that concurrent treatment with infliximab suppresses such resistance in association with inhibition of ERK activation and c-Myc expression. Given that ERK and c-Myc are also downstream mediators of TNF-α signaling and that their sustained activation is related to osimertinib resistance^34,35^, the increased production of TNF-α induced by interaction of mutant p53 with p65 likely contributes to acquisition of osimertinib resistance in *TP53*-GOF mutant cells.

GOF mutant forms of p53 promote the development of severe chronic inflammation and persistent tissue damage that give rise to inflammation-associated colon cancer in a mouse model^36^. Although RELA (p65) has previously been implicated in EGFR-TKI resistance^37,38^ and *TP53*-GOF mutations have previously been found to promote cancer development through enhancement of NF-κB signaling induced by TNF-α stimulation^36,39,40^, the relation of *TP53*-GOF mutations to the efficacy of EGFR-TKI treatment has remained unknown. We have now shown that *TP53*-GOF mutations promote the interaction of p53 with p65, increase TNF-α production, and accelerate the development of resistance to osimertinib in osimertinib-treated cells.

GOF mutant forms of p53 have been shown to bind to other transcription factors and thereby to regulate transcription of their target genes^41,42^. Such binding has also been shown to influence the interaction of regulatory molecules with these transcription factors^20^. Whereas the detailed mechanism underlying the increased binding of p53-GOF mutants to p65 and nuclear translocation of the resulting p53-p65 complexes remains to be characterized, such mutant proteins might activate p65 by influencing molecules that contribute to regulation of the transcriptional activity of p65 such as the NF-κB subunit p50 and the inhibitory protein IκB.

Inhibition of EGFR signaling has been shown to increase the stability of *TNF* mRNA through suppression of miR-21 and to promote TNF-α-induced NF-κB activation, resulting in the development of EGFR-TKI resistance^9^. We have now shown that osimertinib treatment increases *TNF* expression to a much greater extent in *TP53*-GOF mutant cells compared with cells without such mutations. Concurrent treatment with infliximab suppressed the development of resistance to osimertinib promoted by *TP53*-GOF mutations as well as restored osimertinib sensitivity in resistant cells. These findings indicate that upregulation of TNF-α expression as a mechanism of resistance to osimertinib is a characteristic of *TP53*-GOF mutant cells.

Although clinical trials of monotherapy with antibodies to TNF-α for various types of cancer have not demonstrated significant efficacy^43–46^, our findings now suggest that combination therapy with such antibodies and osimertinib is a promising strategy for patients with NSCLC positive for both *EGFR* and *TP53*-GOF mutations. Whereas genomic testing with next-generation sequencing panels has revealed several mechanisms of resistance to osimertinib^35^, the mechanisms responsible for most cases of such resistance remain uncharacterized. Our results now implicate positive feedback signaling dependent on TNF-α production in cancer cells with *TP53*-GOF mutations as a target for overcoming resistance to osimertinib treatment.

Our in vivo experiments revealed a greater increase in *TNF* mRNA abundance (>1000-fold increase) in *TP53*-GOF mutant cells treated with osimertinib compared with that observed in our in vitro experiments (~200-fold increase), suggesting that the tumor microenvironment including macrophages and fibroblasts might contribute to this in vivo effect. In addition, comprehensive RT-qPCR analysis revealed upregulation of the expression of genes for several cytokines or growth factors—including *FGF1*, *NGF*, *IL1A*, *IL11*, *IL8*, and *TGFA*—in PC9/p53^R248Q^ cells compared with PC9/p53^EV^ or PC9/p53^WT^cells (Supplementary Fig. 5). These molecules have been associated with tumor growth or drug resistance^47–49^. Whereas treatment with osimertinib resulted in a decrease in the expression of most of these genes in vitro, it remains possible that the encoded proteins exert effects on the tumor itself or the tumor microenvironment in vivo that contribute to the development of osimertinib resistance.

The FLAURA2 trial showed that the addition of chemotherapy to osimertinib resulted in a significant improvement in therapeutic efficacy for *EGFR*-mutated NSCLC, making this combination a promising option for first-line treatment^50^. However, most patients who received the combination treatment still experienced disease progression within 2 years, underscoring the importance both of the identification of the precise mechanisms of resistance and the development of corresponding therapeutic strategies. It is possible that upregulation of TNF-α is also associated with resistance to the combination therapy in NSCLC with *TP53*-GOF mutations and that anti-TNF-α treatment might be beneficial for such resistant tumors.

There are several limitations to our study. First, although we investigated several types of *TP53*-GOF mutation, our findings cannot be extrapolated directly to all such mutations, given that the different mutation types show specific functional characteristics^42,51^. According to a database^24^, the estimated frequencies of the three GOF mutations examined in our study—R248Q, R273H, and R175H—in patients with advanced NSCLC positive for activating mutations (L858R or exon-19 deletions) of *EGFR* are 0.80%, 0.91%, and 1.26%, respectively. Further investigation to test whether our findings are applicable to other types of *TP53*-GOF mutation is warranted in order to determine whether additional patients might benefit from anti-TNF-α treatment. Second, although expression of the WT p53 protein is usually suppressed by MDM2-dependent degradation, the expression level of p53 in cells stably expressing *TP53*-WT in our study was as high as that in cells stably expressing *TP53*-GOF, consistent with previous observations^28,52^. In addition, although the expression of p21 is usually induced in cells harboring WT p53, such expression was not apparent in cells stably expressing *TP53*-WT in our study. It is possible that the process of establishing cell lines with stable expression of p53 influenced the activity of MDM2 and the expression of p21, and that our finding of *TP53*-GOF mutation-specific resistance to osimertinib induced by the interaction of p53 with p65 is independent of MDM2 and p21. Finally, whereas EGFR-TKIs show a poor efficacy for NSCLC positive for *EGFR* mutations in association with *TP53* mutations^14,15^, whether *TP53*-GOF mutations confer an inferior efficacy for osimertinib in comparison with non-GOF mutations is unclear. Further clinical investigation of the effects of *TP53*-GOF mutations in NSCLC positive for *EGFR* mutations is warranted.

In conclusion, our study provides new insight into a mechanism of osimertinib resistance attributable to *TP53*-GOF mutations and suggests that TNF-α inhibition might be an effective strategy to overcome osimertinib resistance in patients with *EGFR* mutation-positive NSCLC with coexisting *TP53*-GOF mutations.

## Methods

### Cell lines and reagents

Five human NSCLC cell lines with *EGFR* activating mutations (PC-9 [ECACC #90071810], H1975 [ATCC #CRL-5908], II-18 [#RCB2093; RIKEN BioResource Research Center, Tsukuba, Japan], HCC827 [ATCC #CRL-2868], and HCC4006 [ATCC #CRL-2871]) and one human NSCLC cell line WT for *EGFR* (A549 [ATCC #CCL-185]) were studied. The *EGFR* and *TP53* mutation status of each cell line is provided in Supplementary Table 1. PC-9, H1975, II-18, HCC827, and HCC4006 cells were cultured in RPMI 1640 medium (Gibco, Carlsbad, CA, USA), and A549 cells were maintained in DMEM (Gibco). Each medium was supplemented with 10% fetal bovine serum and 1% penicillin-streptomycin (Gibco). All cells were maintained under a humidified atmosphere of 5% CO_2_ and at 37 °C. Osimertinib (#S7297; Selleck Chemicals, Houston, TX, USA) was dissolved in DMSO (Fujifilm Wako Pure Chemical Industries, Osaka, Japan) and stored at −20 °C. Infliximab (Mitsubishi Tanabe Pharma, Osaka, Japan) and doxycycline (#631311; Takara Bio, Shiga, Japan) were stored at −20 °C. JSH-23 (#S7351, Selleck Chemicals) was dissolved in DMSO (Fujifilm Wako Pure Chemical Industries) and stored at −20 °C. Recombinant human TNF-α (#210-TA-005; R&D Systems, Minneapolis, MI, USA) was dissolved in sterile phosphate-buffered saline (PBS) containing 0.1% bovine serum albumin and was stored at −20 °C.

### Establishment of p53-KO cell lines

Disruption of *TP53* in PC-9, H1975, and HCC4006 cells was performed with the CRISPR-Cas9 system. The crRNA was designed with the use of the CRISPR-Cas9 guide RNA design checker (https://sg.idtdna.com/site/order/designtool/index/CRISPR_SEQUENCE) and was synthesized by Integrated DNA Technologies (Coralville, IA, USA). It was targeted to the sequence 5′-CCATTGTTCAATATCGTCCG-3′ at position 7,676,209 of the plus strand and with a protospacer adjacent motif (PAM) of GGG. Guide RNA was prepared by mixing crRNA and tracrRNA (#1072532, IDT), and 1.5 μl of the guide RNA oligonucleotides, 1.5 μl of 1 μM Cas9 (#1081058, IDT), and 22.0 μl of Opti-MEM (#31985-062, Gibco) were then combined and incubated for 5 min at room temperature to allow formation of the ribonucleoprotein complex, to which was then added 1.2 μl of RNAiMAX (#13778100; Thermo Fisher Scientific, Waltham, MA, USA) and 23.8 μl of Opti-MEM in order to generate the transfection complex. Cells (4 × 10^4^) suspended in 50 μl of RPMI 1640 medium were added together with the transfection complex (50 µl) to individual wells of a 96-well plate and incubated for 48 h before passage. Single cells were sorted from the transfected cells by fluorescence-activated cell sorting with the use of a FACS Aria instrument (BD Biosciences, Franklin Lakes, NJ, USA) and were then transferred individually to the wells of a 96-well plate and cultured at 37 °C for 14 to 21 days. Tracking of indels by decomposition (TIDE) analysis (https://tide.nki.nl) and immunoblot analysis were performed to identify p53-KO clones. Single-cell clones showing complete loss of p53 protein (PC9/p53^KO^, H1975/p53^KO^, and HCC4006/p53^KO^ cells) were established.

### Plasmid construction

Complementary DNA for WT *TP53* was obtained from A549 cells, and that for mutant *TP53* (R248Q, R273H, V218del, or Y205H) from PC-9, H1975, HCC827, and HCC4006 cells, respectively, with the use of PrimeScript RT Master Mix (#RR036A, Takara Bio). Corresponding PCR amplicons were then prepared with the use of PrimeSTAR GXL DNA Polymerase (#R050A, Takara Bio) and specific primers (Supplementary Table 3). The R175H mutation of *TP53* was generated by overlap-extension PCR with forward (5′-TTGTGAGGCACTGCCCCCACCATGAGCGCTG-3′) and reverse (5′-GGCAGTGCCTCACAACCTCCGTCATGTGC-3′) primers designed to introduce the specified base change (c.524G > A), as previously described^53^. The coding sequences for the WT and mutant p53 proteins were verified by Sanger sequencing and were then ligated into the pQCXIP retroviral vector (#639648; Clontech, Kusatsu, Japan) between the NotI and BamHI sites with the use of an In-Fusion HD Cloning Kit (#639648, Takara Bio). For construction of *TP53* plasmids for the Tet-On system, PCR products were generated with the primers listed in Supplementary Table 3 and ligated into the pTetOne retroviral vector (Takara Bio) between the EcoRI and BamHI sites with the use of an In-Fusion HD Cloning Kit.

### Retrovirus transduction for generation of stable cell lines

For establishment of cell lines stably expressing WT or mutant forms of p53, the constructed plasmid vectors described above or the empty plasmid vector (EV) were first introduced into HEK293T cells (ATCC #CRL-1573) with the use of a Retrovirus Packaging Kit Ampho (#6161, Takara Bio) and the Lipofectamine 3000 reagent (Invitrogen, Carlsbad, CA, USA). The culture supernatants containing the recombinant retroviruses were then passed through a 0.45-μm filter, and the filtrate was incubated overnight at 4 °C with a Retro-X Concentrator (Clontech, Shiga, Japan) and then centrifuged at 1500 × *g* for 45 min at 4 °C for isolation of virus pellets. PC9/p53^KO^, HCC4006/p53^KO^, or H1975/p53^KO^ cells were infected with the retroviruses for 24 h in the presence of polybrene (Nacalai Tesque, Kyoto, Japan) at 8 μg/ml and were then cultured in growth medium for an additional 24 h before selection by culture in the presence of puromycin (Invitrogen) at 1 μg/ml. For establishment of cell lines for doxycycline-inducible p53 expression, GP2-293 packaging cells of the Retro-X Tet-One Inducible Expression System (#634307, Takara Bio) were used to produce retroviruses instead of HEK293T cells.

### Cell viability assay

Cells (3000 per well) were seeded in a 96-well flat-bottom plate (Greiner Bio-One, Kremsmunster, Austria) and incubated at 37 °C under 5% CO_2_ for 24 h before exposure to test agents and incubation for an additional 72 h. Cell Counting Kit 8 (Nacalai Tesque) reagent (10 μl) was then added to each well, the cells were incubated for an additional 2 h, and absorbance at 450 nm was measured with a Multiskan FC instrument (Thermo Fisher Scientific).

### Induction of osimertinib resistance

For generation of osimertinib-resistant cell lines and examination of the development of osimertinib resistance, we adopted two different experimental protocols. First, PC9/p53, HCC4006/p53, or H1975/p53 cells (1.2 × 10^5^ per well) were seeded in 12-well plates (Greiner Bio-One) and treated with 1 μM or 600 nM osimertinib for 28 days, and the number of cells per well was counted with a LUNAII automated cell counter (Logos Biosystems, Gyeonggi-do, Republic of Korea) at the indicated times during drug treatment. Second, PC9/p53 cells (1.2 × 10^5^ per well) were seeded in six-well plates (Greiner Bio-One) and exposed to increasing concentrations of osimertinib from 10 nM to 1 μM over a maximum of 30 days, with the osimertinib concentration being increased when the cells had achieved 70% confluence. PC9/p53^R248Q^, PC9/p53^R273H^, and PC9/p53^R175H^ cells that acquired resistance to 1 μM osimertinib were defined as osimertinib-resistant cells and studied further.

### Immunoblot analysis

Cells were washed with ice-cold PBS and then lysed in RIPA buffer (Thermo Fisher Scientific) containing protease and phosphatase inhibitors (Nacalai Tesque). The lysates were fractionated by SDS-polyacrylamide gel electrophoresis on a 10% gel, and the separated proteins were transferred to a polyvinylidene difluoride membrane. The membrane was incubated overnight at 4 °C with primary antibodies to phospho-NF-κB p65 (#3033), to NF-κB p65 (#8242), to phospho-EGFR (#3777), to EGFR (#4267), to phospho-ERK1/2 (#4370), to ERK1/2 (#9102), to phospho-AKT (#9271), to AKT (#9272), to c-Myc (#5605), to p53 (#2527), to p21 (#2947), to HER2 (#2242), to MET (#8198), or to β-actin (#4970), all of which were obtained from Cell Signaling Technology (Danvers, MA, USA) and used at a dilution of 1:1000. The membrane was subsequently incubated for 1 h at room temperature with horseradish peroxidase-conjugated antibodies to rabbit IgG (#NA9340, diluted 1:10,000; Cytiva, Tokyo, Japan), after which immune complexes were detected with the use of Pierce ECL Plus Immunoblotting Substrate (Thermo Fisher Scientific) and images were captured with a ChemiDoc Touch MP system (Bio-Rad, Hercules, CA, USA). All blots were derived from the same experiments and were processed in parallel. Uncropped scans of the most important blots are provided as Supplementary Fig. 10 in the Supplementary Information.

### RNA-seq analysis

Total RNA was isolated with the use of an RNeasy Mini Kit (#74016; Qiagen, Hilden, Germany) from cells that had been treated with 600 nM osimertinib or DMSO vehicle for 24 h. Two biological replicates were used for each condition. The quantity and quality of the RNA were determined with the use of a NanoDrop-2000 spectrophotometer (Thermo Fisher Scientific) and a 2200 TapeStation (Agilent Technologies, Santa Clara, CA, USA), respectively, and rRNA was removed with an MGI Easy rRNA Depletion Kit (MGI, Shenzhen, China) before library construction with an MGI Easy RNA Directional Library Prep Set (MGI). Deep sequencing of amplicons was performed with an MGI DNBseq-G400 FAST instrument. The sequence format was 150-bp paired reads for all samples. All sequencing reads were trimmed of low-quality bases and adapters with the use of Trimmomatic (version 0.38) (Bolger AM, Golm, Germany), and RNA-seq reads were mapped to the hg38 genome with the use of HISAT2 software (version 2.1.0). Raw counts for each gene were estimated for each sample with RSEM version 1.3.0 and Bowtie 2. Calculation of the log_2_[fold change] and *p* values was performed with edgeR (R Bioconductor). Gene enrichment analysis was performed with the use of Metascape^54^.

### RT-qPCR analysis

Total RNA was extracted from cell lines and tumors of the mouse model with the use of an RNeasy Mini Kit (Qiagen) and was subjected to RT with a PrimeScript RT Reagent Kit (#RR037A, Takara Bio). The resulting cDNA was subjected to qPCR analysis with SYBR Green PCR Master Mix (#4344463, Thermo Fisher Scientific) and specific primers (Supplementary Table 4). Relative expression of the target genes was analyzed with the 2^–ΔΔCt^ method and was normalized by *GAPDH* mRNA abundance. A *z*-score-based heat map for hierarchical clustering was generated with Heatmapper (http://www.heatmapper.ca).

### ELISA

For measurement of TNF-α levels in culture supernatants, cells were cultured in serum-free medium and treated with 600 nM osimertinib for 48 h. Culture supernatants were then collected and were concentrated with the use of an Amicon Ultra centrifugal filter (#503012; Millipore, Billerica, MA, USA), and the concentration of TNF-α was measured with an AuthentiKine TNF-α ELISA Kit (#KE00154; Proteintech, Rosemont, IL, USA).

### ChIP-qPCR analysis

Prediction of the p65 binding site in the *TNF* promoter region was based on the JASPAR database (http://jaspar.genereg.net), as shown previously^55^. PC9/p53^V218del^, PC9/p53^WT^, PC9/p53^R248Q^, PC9/p53^R273H^, and PC9/p53^R175H^ cells were incubated with 600 nM osimertinib or DMSO vehicle for 24 h, after which DNA-protein complexes were isolated with the use of a ChIP Assay Kit (#17-295, Millipore), as previously described^56^. In brief, the cells were first incubated with 4% formaldehyde for 10 min at 37 °C to induce cross-linking, and were then subjected to ultrasonic treatment to shear cross-linked DNA into fragments of ~200 to 500 bp with a Bioruptor UCD-300 device (Cosmo Bio, Tokyo, Japan). DNA-protein complexes were immunoprecipitated with antibodies to p65 (#8242) or to p53 (#2527), or with control rabbit IgG (#2729), all of which were obtained from Cell Signaling Technology. DNA was purified from the precipitates with the use of a QIAquick PCR Purification Kit (#28104, Qiagen) and was then subjected to qPCR analysis with primers specific for the *TNF* promoter and control regions (Supplementary Table 5).

### ChIP-seq analysis

PC9/p53^R248Q^ cells were incubated with 600 nM osimertinib for 0, 24 h, or 1 week. The same method as used in the ChIP-qPCR assay was used for DNA-protein complexes isolation. DNA-protein complexes were immunoprecipitated with antibodies to p53 (#2527, Cell Signaling Technology). DNA was purified from the precipitates with the use of a QIAquick PCR Purification Kit. ChIP-seq libraries were prepared using ThruPLEX DNA-seq Kit (#RB4677, Takara Bio) according to the manufacturer’s instructions. Sequencing was performed using 150-bp paired-end reads on the MGI DNBSEQ-G400 genome sequencer. Read quality was evaluated with FastQC (Version 0.11.7), and after low-quality (<20) bases and adapter sequences were trimmed using Trimmomatic, they were aligned to the reference genome using Bowtie2. Peaks were called using the MACS2 (Version 2.1.2)^57^ and were merged with Bedtools (Version 2.27.1)^58^. The abundance of uniquely mapped reads was estimated with featureCounts (Version 1.6.3)^59^. The raw read counts were normalized by the Trimmed mean of M values (TMM), and differential analysis was conducted with edgeR. Differential peak regions were detected with the thresholds of | log_2_[fold change] | > 1 and FDR < 0.05 by the Benjamini–Hochberg method. Motif discovery was performed by HOMER (Version 4.1)^60^.

### In situ PLA

PC9/p53^EV^, PC9/p53^WT^, and PC9/p53^R248Q^ cells were cultured to 70% confluence on 12-mm-diameter coverslips (Matsunami, Osaka, Japan) placed in 24-well plates (Corning, Corning, NY). PC9/p53^R248Q^ cells were treated with either DMSO as a control, 600 nM osimertinib, or TNF-α (1 ng/ml) for 24 h. Cells were fixed for 15 min with 4% paraformaldehyde in PBS, permeabilized for 10 min with 0.3% Triton X-100 in PBS, and incubated overnight at 4 °C with 1:500 dilutions of mouse antibodies to p53 (#2524, Cell Signaling Technology) and rabbit antibodies to p65 (#8242, Cell Signaling Technology) for detection of p53-p65 complexes with the use of Duolink PLA Fluorescence Kits (#DUO92002 and #DUO92004; Sigma-Aldrich, St. Louis, MO, USA). Images of PLA signals were obtained with a BZX800 all-in-one fluorescence microscope (Keyence, Osaka, Japan). Optical sectioning images of p53-p65 complexes in multiple cells acquired from top to bottom of each cell were combined into a *z*-projection image with the use of full-focus imaging (BZ-H4A, Keyence). The number of PLA signals per cell and percentage of the signals in the nucleus stained with DAPI were quantified in nine fields including a total of at least 50 cells for each condition with the use of BZ-X Analyzer software (BZ-H4A, Keyence).

### Animal studies

Four-week-old female athymic mice were obtained from CLEA Japan (Tokyo, Japan). PC9/p53^EV^ or PC9/p53^R248Q^ cells (5.0 × 10^6^) were injected subcutaneously into the flank of the mice, which were randomly divided into the treatment groups described in Fig. 7 at 10 days after cell injection. Tumor dimensions were measured twice each week, and tumor volume was calculated according to the formula: (length × width × width)/2. Mice were killed by cervical dislocation under anesthesia when tumors achieved a volume of >2000 mm^3^ or 30 days after treatment onset. For RNA extraction, tumors were isolated 14 days after treatment onset.

### Statistics

Data are presented as means ± SEM or ± SD and were compared with the unpaired Student’s *t* test, the Mann–Whitney test, or one-way analysis of variance (ANOVA) followed by Tukey’s test as performed with GraphPad Prism 9 software. A *p* value of <0.05 was considered statistically significant.

### Study approval

Animal experiments were approved by the Kyushu University Animal Experiment Committee (approval number: A22-379-1) and were performed in accordance with Kyushu University Animal Experiment Regulations, related laws and regulations, and ARRIVE (Animal Research: Reporting of In Vivo Experiments) guidelines.

### Reporting summary

Further information on research design is available in the Nature Research Reporting Summary linked to this article.

### Supplementary information

Supplementary information

Reporting summary

## Data Availability

All RNA-seq and ChIP-seq data sets have been deposited, and processed values and complete gene lists are available at GEO (http://www.ncbi.nlm.nih.gov/geo) under GEO series IDs GSE232890 and GSE253478, respectively.
