# Supplementary material for: TP53 gain-of-function mutations promote osimertinib resistance via TNF-α–NF-κB signaling in EGFR-mutated lung cancer
Source: NPJ Precis Oncol. 2024 Mar 2;8:60. doi: 10.1038/s41698-024-00557-2 (PMC10908812; doi:10.1038/s41698-024-00557-2)
Supplement: Supplementary file 1 — Supplementary information [file 41698_2024_557_MOESM1_ESM.pdf]

Supplemental Fig. 1

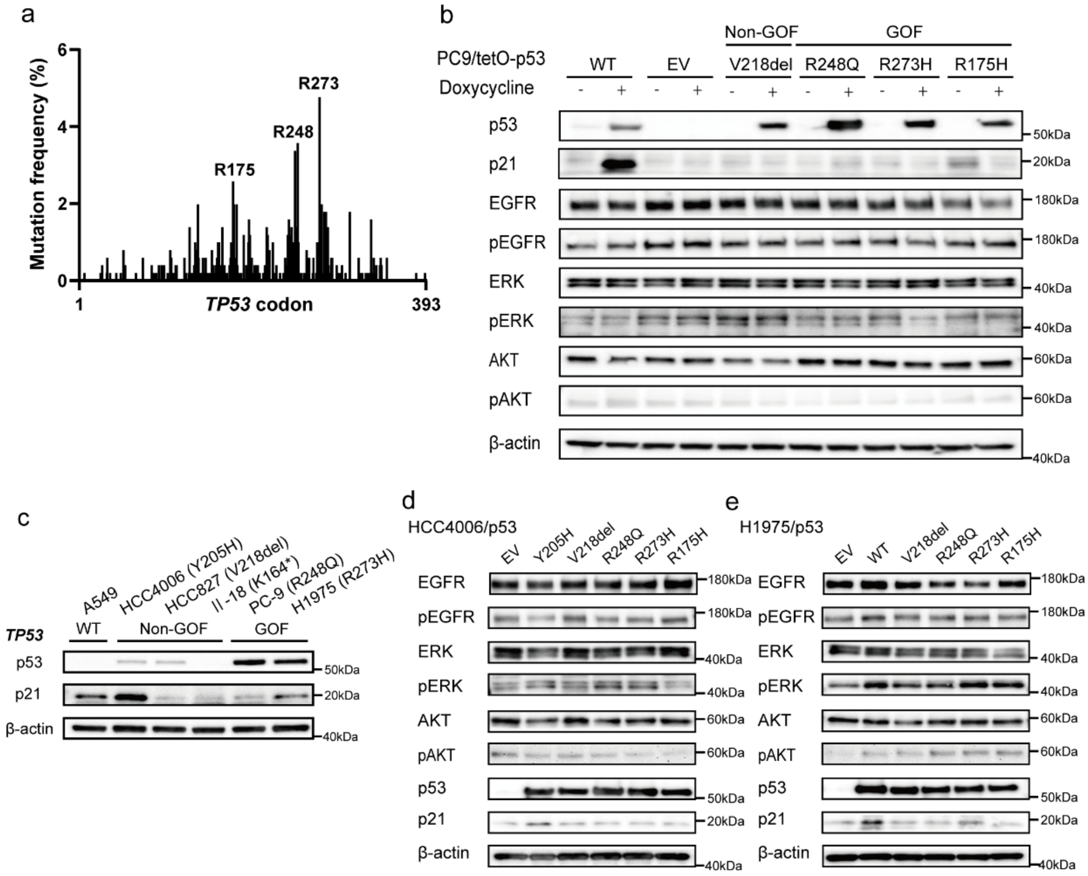

Supplementary Fig. 1 *TP53* mutation in lung cancer. a Frequency of *TP53* mutations at each codon of the gene in patients with advanced NSCLC positive for both *EGFR* activating mutations (exon-19 deletions or L858R point mutation) and *TP53* mutations (n = 504) as determined from data of the MSK-MET study accessed via the cBioPortal database. **b** Immunoblot analysis of p53 and p21 expression as well as of EGFR signaling in PC9/tetO-p53^{EV}, PC9/tetO-p53^{WT}, and PC9/tetO-p53^{MUT} cells incubated in the absence or presence of doxycycline (1 µg/mL) for 24 h. *TP53* mutant status is classified as non-GOF or GOF. **c** Immunoblot analysis of p53 and p21 in various human NSCLC cell lines classified according to *TP53* status. **d**, **e** Immunoblot analysis of EGFR signaling as well as of p53 and p21 in HCC4006/p53^{EV} and HCC4006/p53^{MUT} cells (**d**) and in H1975/p53^{EV}, H1975/p53^{WT}, and H1975/p53^{MUT} cells (**e**).

Supplemental Fig. 2

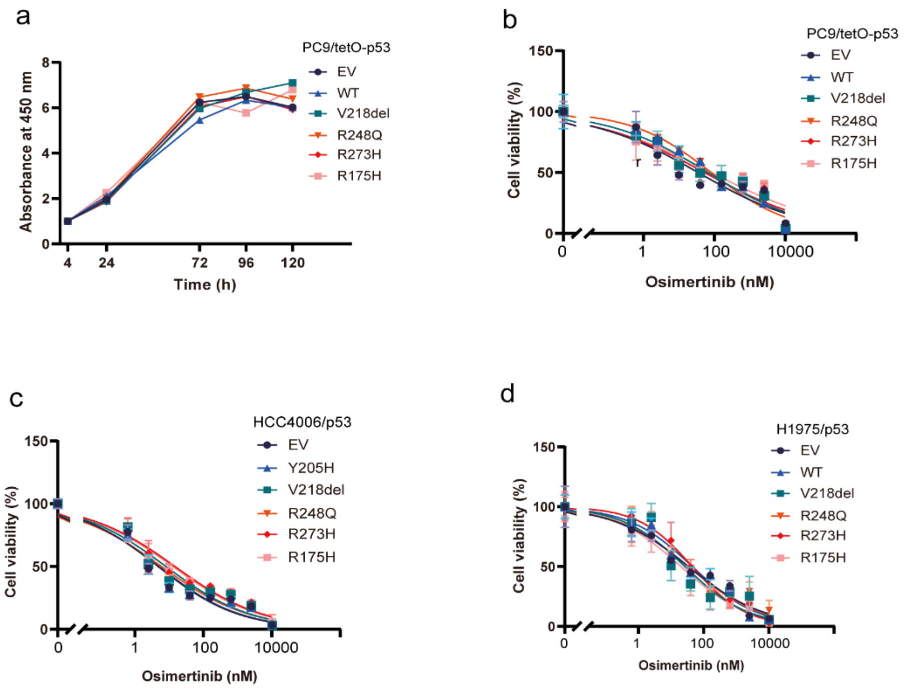

Supplementary Fig. 2 *TP53* status does not affect osimertinib sensitivity of *EGFR*-mutant NSCLC cells. a Cell proliferation curves for PC9/tetO-p53^{EV}, PC9/tetO-p53^{WT}, and PC9/tetO-p53^{MUT} cells measured with a colorimetric assay. **b–d** Viability of PC9/tetO-p53^{EV}, PC9/tetO-p53^{WT}, and PC9/tetO-p53^{MUT} cells (**b**), HCC4006/p53^{EV} and HCC4006/p53^{MUT} cells (**c**), and H1975/p53^{EV}, H1975/p53^{WT}, and H1975/p53^{MUT} cells (**d**) treated with the indicated concentrations of osimertinib for 72 h as evaluated with a colorimetric assay. In both **a** and **b**, the cells were exposed to doxycycline (1 µg/mL) for 24 h before and during the experimental incubation. All data are means ± SEM for triplicates from one experiment in each case and are representative of three independent experiments.

Supplemental Fig. 3

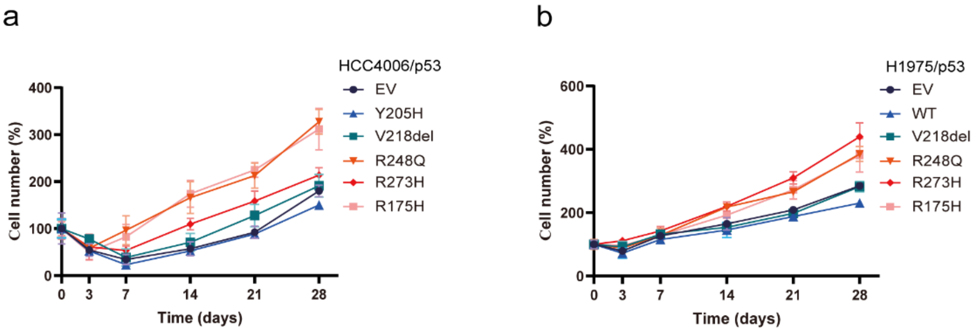

Supplementary Fig. 3 *TP53*-GOF mutation promotes the early development of osimertinib resistance in HCC4006 and H1975 cells. Time courses of HCC4006/p53^{EV} and HCC4006/p53^{MUT} (**a**) as well as H1975/p53^{EV}, H1975/p53^{WT}, and H1975/p53^{MUT} (**b**) cell number during treatment with 600 nM osimertinib (**a**) or 1 μM osimertinib (**b**) for up to 28 days are shown. Data are means ± SEM for triplicates from one experiment in each case and are representative of three independent experiments.

Supplemental Fig. 4

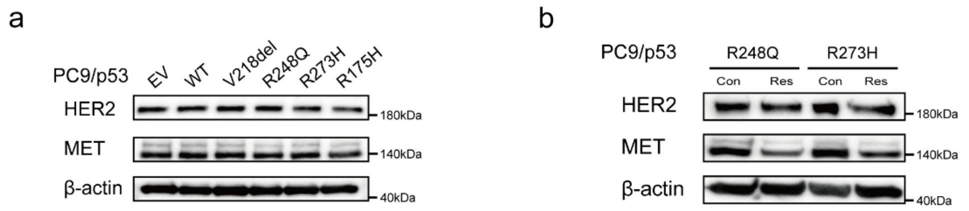

Supplementary Fig. 4 Immunoblot analysis of HER2 and MET. PC9/p53^{EV}, PC9/p53^{WT}, and PC9/p53^{MUT} cells under basal conditions (**a**) as well as PC9/p53^{R248Q} and PC9/p53^{R273H} cells either not exposed to osimertinib (Con) or with acquired resistance to osimertinib induced by long-term exposure to the drug (Res) (**b**) were subjected to immunoblot analysis of HER2 and MET.

Supplemental Fig. 5

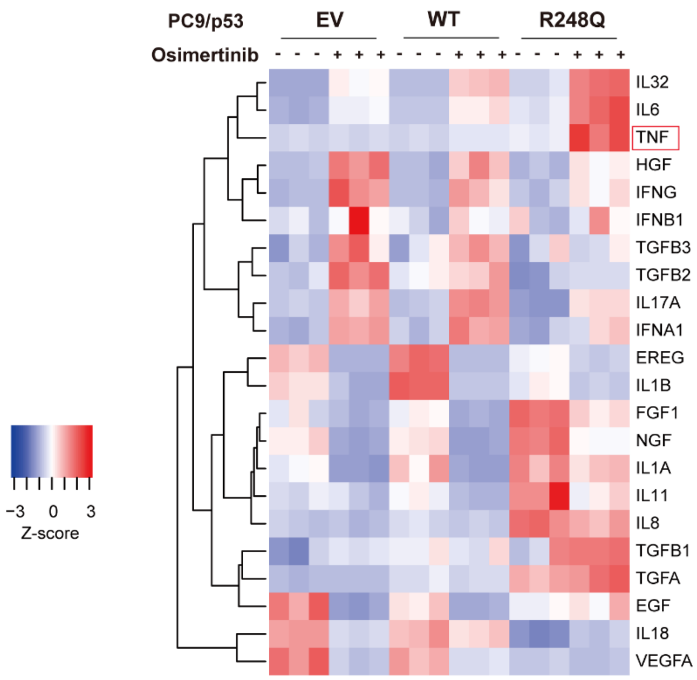

Supplementary Fig. 5 Heat map for hierarchical clustering of cytokine gene expression. PC9/p53^{EV}, PC9/p53^{WT}, and PC9/p53^{R248Q} cells were incubated in the absence or presence of 600 nM osimertinib for 24 h before RT-qPCR analysis of mRNA abundance for various cytokines implicated in tumor progression. The heat map shows the *z*-score according to the indicated color scale. Data are shown for triplicates from one experiment and are representative of two independent experiments.

Supplemental Fig. 6

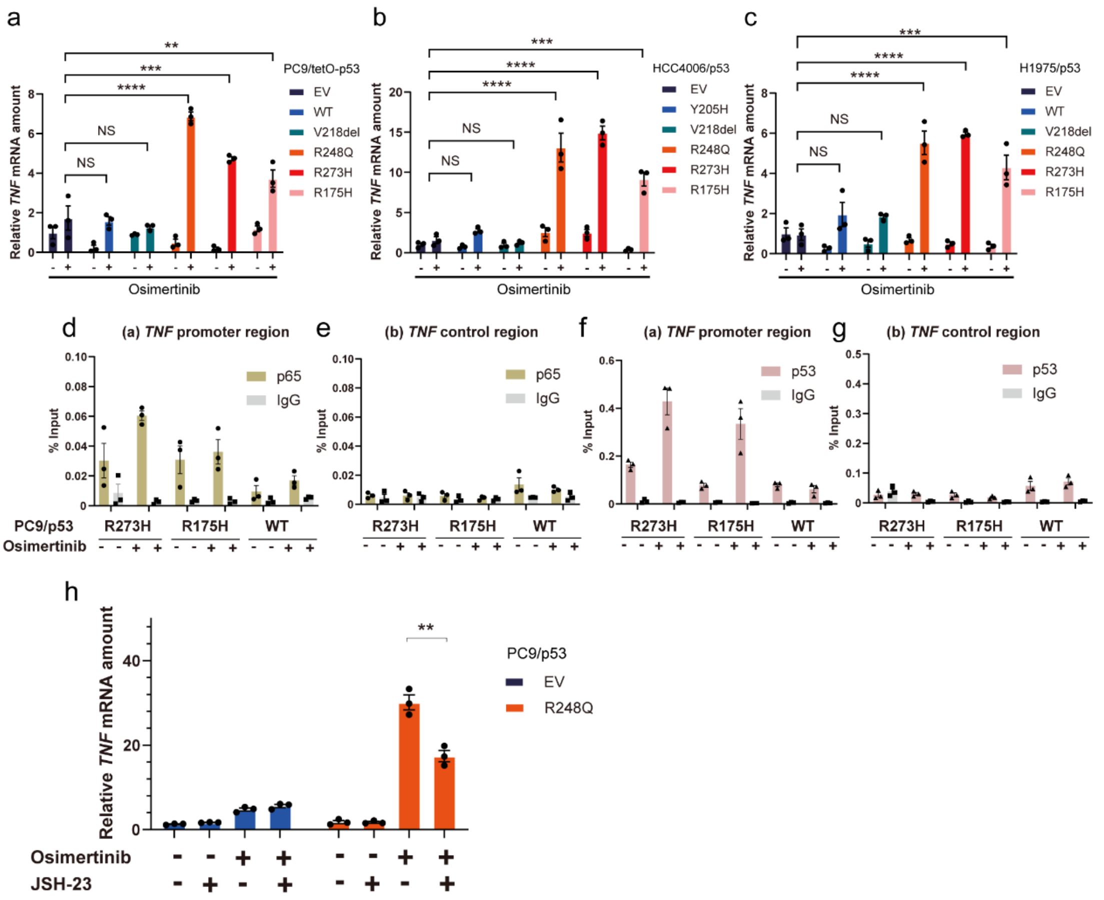

Supplementary Fig. 6 *TP53*-GOF mutation promotes *TNF* expression in osimertinib-treated cells in a manner dependent on p53-p65 interaction. a–c PC9/tetO-p53^{EV}, PC9/tetO-p53^{WT}, and PC9/tetO-p53^{MUT} cells (**a**), HCC4006/p53^{EV} and HCC4006/p53^{MUT} cells (**b**), and H1975/p53^{EV}, H1975/p53^{WT}, and H1975/p53^{MUT} cells (**c**) were incubated in the absence or presence of 600 nM osimertinib for 24 h before RT-qPCR analysis of *TNF* mRNA abundance. **d–g** Percentage of ChIP-qPCR amplicons derived from the promoter (**d**, **f**) or control (**e**, **g**) regions of *TNF* that were immunoprecipitated with antibodies to p65 (**d**, **e**) or to p53 (**f**, **g**), or with control IgG, from PC9/p53^{WT}, PC9/p53^{R273H}, or PC9/p53^{R175H} cells incubated with or without 600 nM osimertinib for 24 h. ChIP-qPCR primers were designed to amplify promoter (a) or control (b) regions of the *TNF* gene locus shown in Figure 4a. **h** PC9/p53^{EV} and PC9/p53^{R248Q} cells were incubated in the absence or presence of 600 nM osimertinib and 10 μM JSH-23 for 24 h before RT-qPCR analysis of *TNF* mRNA abundance. All data are means ± SEM of triplicates from one experiment in each case and are representative of three independent experiments. ***P* < 0.01, ****P* < 0.001, *****P* < 0.0001, NS (one-way ANOVA followed by Tukey's test).

Supplemental Fig. 7

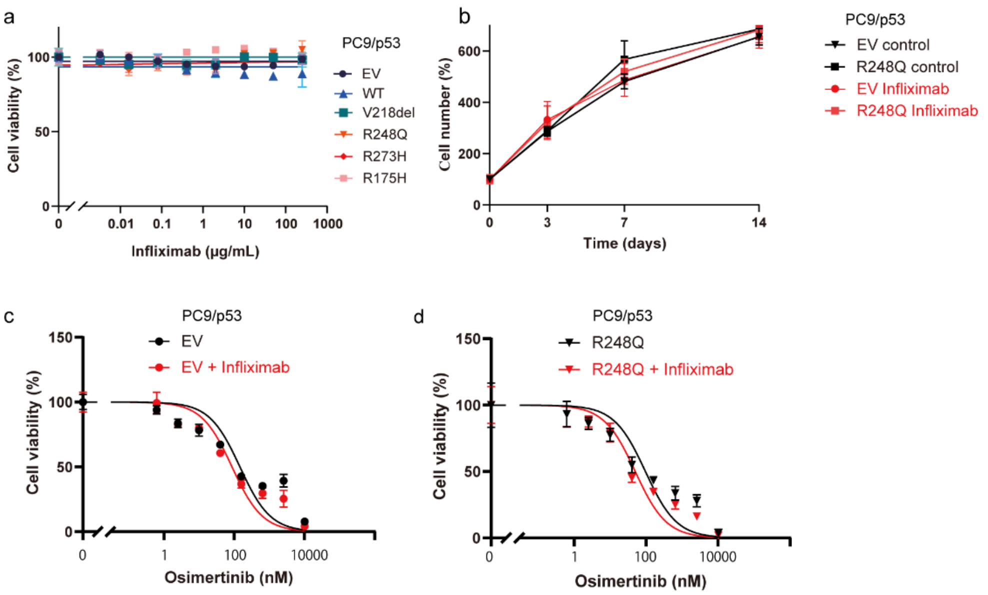

Supplementary Fig. 7 *TP53* status does not affect the sensitivity of *EGFR*-mutant NSCLC cells to infliximab. a Viability of PC9/p53^{EV}, PC9/p53^{WT}, and PC9/p53^{MUT} cells treated with the indicated concentrations of infliximab for 72 h as evaluated by a colorimetric assay. **b** Percentage cell number for PC9/p53^{EV} and PC9/p53^{R248Q} cells treated with DMSO as a control or infliximab (1 µg/mL) for the indicated times. **c**, **d** Viability of PC9/p53^{EV} (**c**) and PC9/p53^{R248Q} (**d**) cells treated with the indicated concentrations of osimertinib in the absence or presence of infliximab (1 µg/mL) for 72 h. All data are means ± SEM of triplicates from one experiment in each case and are representative of three (**a**, **c**, **d**) or two (**b**) independent experiments.

Supplemental Fig. 8

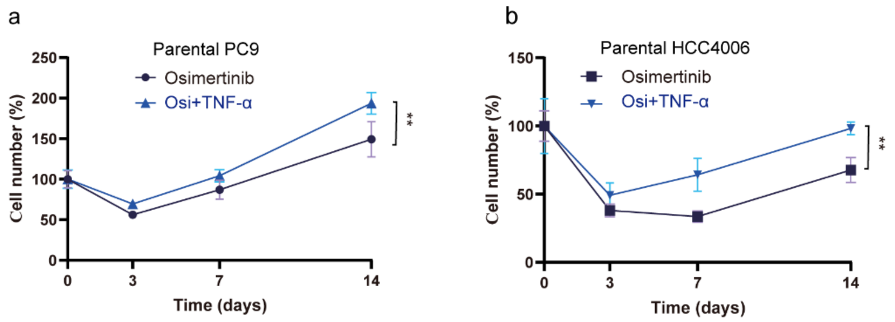

Supplementary Fig. 8 TNF-α promotes osimertinib resistance in parental cell lines. Percentage cell number is shown for parental PC9 (**a**) and parental HCC4006 (**b**) cells treated for the indicated times with 1 μM or 600 nM osimertinib (Osi), respectively, in the absence or presence of TNF-α (10 pg/mL). Data are means ± SEM of triplicates from one experiment in each case and are representative of two independent experiments. $^{**}P < 0.01$ (one-way ANOVA followed by Tukey's test).

Supplemental Fig. 9

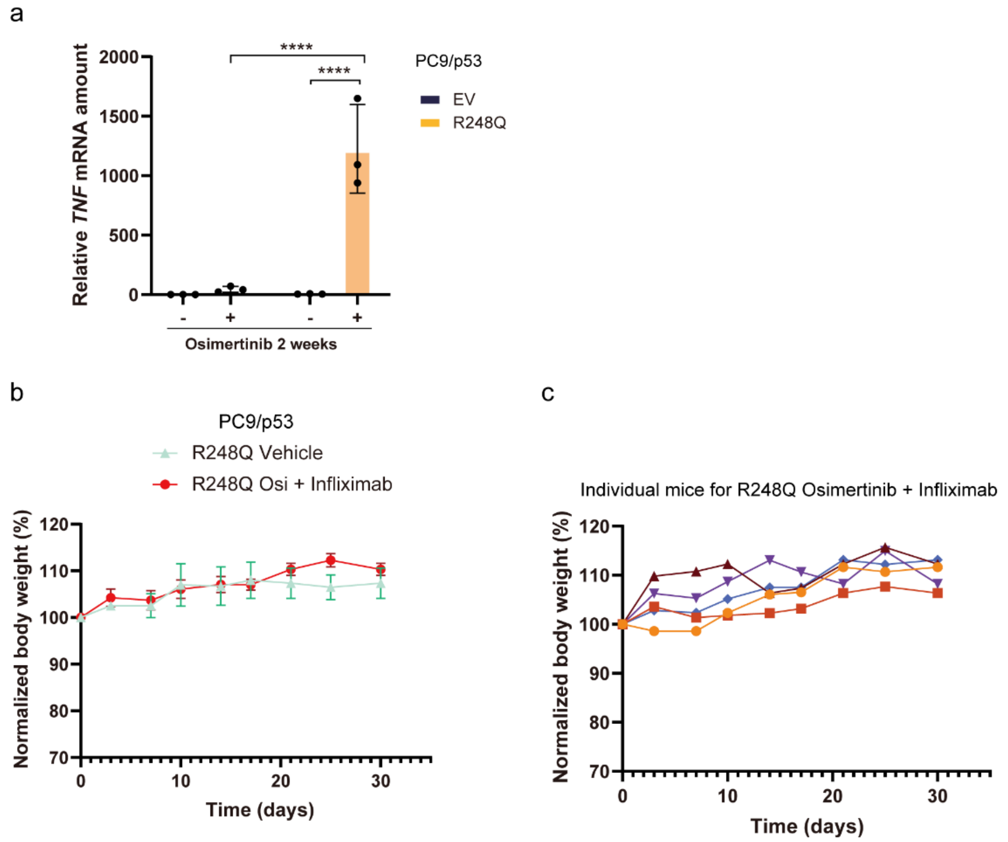

Supplementary Fig. 9 Effects of osimertinib on *TNF* expression and of osimertinib plus infliximab on body weight in a xenograft mouse model. a RT-qPCR analysis of *TNF* mRNA abundance in tumors formed by PC9/p53^{EV} or PC9/p53^{R248Q} cells in nude mice and treated with vehicle or osimertinib for 14 days. Data are means ± SEM ($n = 3$ mice per group). ****$P < 0.0001$, (one-way ANOVA followed by Tukey's test). **b** Time course of body weight for mice during treatment with vehicle or with osimertinib plus infliximab in Figure 7c. Data are means ± SEM ($n = 5$ mice per group). **c** Time course of body weight for each of the five mice treated with osimertinib plus infliximab in Figure 7c.

Supplemental Fig. 10

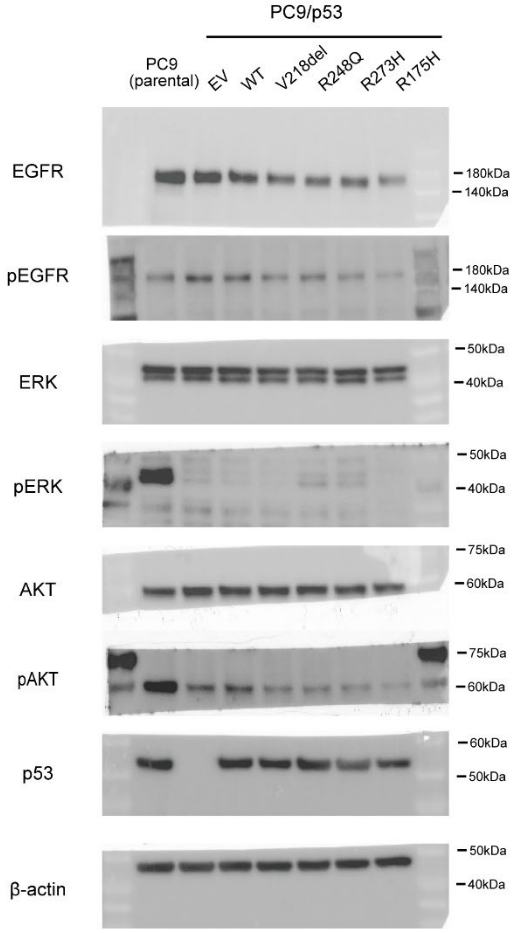

Supplementary Fig. 10 Uncropped scans of blots included in main Figure 1a.

Supplementary Table 1. *TP53* and *EGFR* status of cell lines.

Cell line	*TP53* status	*EGFR* status
A549	WT	WT
HCC4006	Y205H (non-GOF)	Exon-19 deletion
HCC827	V218del (non-GOF)	Exon-19 deletion
Ⅱ-18	K164* (non-GOF)	L858R
PC-9	R248Q (GOF)	Exon-19 deletion
H1975	R273H (GOF)	L858R + T790M

Supplementary Table 2. List of the 111 genes shared between set A and set B in Figure 3a and their log_2[fold change (FC)] values.

Gene symbol	Set A_log_2[FC]	Set B_log_2[FC]	Gene symbol	Set A_log_2[FC]	Set B_log_2[FC]	Gene symbol	Set A_log_2[FC]	Set B_log_2[FC]
RTP4	7.149	1.397	COMMD3-BMI1	2.064	1.391	SLC52A1	1.424	1.892
LGR5	6.677	1.372	DUSP15	2.063	2.498	LOC554249	1.413	6.130
CNTF	6.548	1.910	PSG5	2.063	1.333	MIAT	1.392	1.149
DYX1C1-CCPG1	5.969	5.773	DUSP13	2.046	3.059	KIF17	1.381	1.175
OTOF	5.865	2.443	ADAMTSL2	2.042	2.206	CERS4	1.364	1.998
TMEM189-UBE2V1	5.494	5.299	MX2	2.010	1.159	ARL10	1.360	1.053
SLCO2B1	5.494	2.926	MGC50722	2.004	2.012	PRH1-PRR4	1.344	2.719
ST6GALNAC5	5.180	2.612	C8orf46	1.978	2.289	C4orf26	1.343	1.533
OLR1	3.897	1.017	LRRC43	1.871	1.492	SYNPO2L	1.342	2.569
PSG8	3.826	1.648	TRIM74	1.814	2.199	ADAMTS14	1.328	1.519
FLG	3.274	5.298	KRT34	1.775	1.046	SEPT5-GP1BB	1.307	6.349
CLCNKB	3.269	2.903	FAM225B	1.770	2.002	CSF1R	1.291	2.032
SEPP1	3.225	1.890	DDO	1.769	1.542	FCGRT	1.290	1.955
TMEM130	3.197	1.098	HCST	1.757	1.559	C5AR1	1.282	1.035
CSDC2	3.042	1.187	TMPRSS3	1.750	1.148	LEKR1	1.261	1.168
NR4A3	3.014	2.642	GAL3ST4	1.748	1.247	FMO5	1.255	1.949
CD72	2.960	4.986	FRMPD3	1.729	1.968	GLT8D2	1.248	2.667
TSPY26P	2.957	2.614	RNF150	1.690	1.964	KLK11	1.246	1.548
EFS	2.907	3.401	KCNC3	1.664	2.585	C15orf38-AP3S2	1.242	1.198
PDE6G	2.817	1.497	ADAP2	1.657	2.802	FOLR1	1.234	3.023
CAPN13	2.713	1.767	SYNGR3	1.603	1.168	PON3	1.219	1.903
KCNH3	2.693	3.179	PIFO	1.596	1.775	RCAN2	1.197	2.172
SAA1	2.615	1.175	MAPT	1.589	2.445	RASA4B	1.180	2.144
IL6	2.601	1.223	WFDC21P	1.589	5.668	RASIP1	1.143	1.236
VWA5A	2.566	5.432	IL32	1.575	1.029	GPR173	1.136	1.448
CCDC154	2.440	2.072	SBK2	1.566	1.068	ERICH5	1.106	1.041
MYOZ1	2.341	1.957	LDHD	1.565	1.972	SELM	1.092	1.216
CRYAB	2.314	2.492	ANKRD34B	1.547	1.680	PLEKHG4B	1.084	1.653
THEMIS2	2.293	1.049	GJC3	1.542	1.724	SERPING1	1.078	5.014
PDGFRB	2.287	5.150	LIMS2	1.527	1.114	RBP5	1.071	1.343
NPPB	2.219	2.915	TNFRSF9	1.524	2.710	FKBP1B	1.062	1.983
MAGEC2	2.172	5.555	FCGBP	1.502	2.511	EDN2	1.060	1.402
SSPO	2.140	2.245	PDZD7	1.489	2.506	NPHP3-ACAD11	1.047	1.525
HECW2	2.117	1.022	NGFR	1.465	2.307	PROS1	1.015	3.361
LOC728392	2.100	3.123	STAC2	1.462	3.000	EFEMP2	1.004	1.126
CSF2RB	2.078	1.914	HRNR	1.459	2.628			
ZNRD1-AS1	2.073	2.656	HMGCLL1	1.439	2.036			
PDZK1IP1	2.071	1.324	STON1	1.432	2.835			

Supplementary Table 3. Oligonucleotide sequences for plasmid construction.

Vector	Forward primer (5′-3′)	Reverse primer (5′-3′)
pQCXIP	TTGTGAGGCACTGCCCCCACCATGAGCGCTG	GGCAGTGCCTCACAACCTCCGTCATGTGC
pTetOne	GAGGTGGTCTGGATCTCAGTCTGAGTCAGGCCC	CCCTCGTAAAGAATTATGGAGGAGCCGCAGTCAG

Supplementary Table 4. Oligonucleotide sequences for RT-qPCR analysis of gene expression.

Gene	Forward primer (5′-3′)	Reverse primer (5′-3′)
TNF	GCCCATGTTGTAGCAAACCC	GAGGTACAGGCCCTCTGATG
IL6	ACTCACCTCTTCAGAACGAATTG	CCATCTTTGGAAGGTTCAGGTTG
IL32	GGACGTGGACAGGTGATGTC	CTGTCTCCAGGTAGCCCTCT
IL11	AGCGGACAGGGAAGGGTTA	AGGCGGCAAACACAGTTCAT
NGF	GAGCGCAGCGAGTTTTGG	AGTGTGGTTCCGCCTGTATG
IL17A	CACCTTGGAATCTCCACCGC	GGATCTCTTGCTGGATGGGG
IFNA1	AATACAGCCCTTGTGCCTGG	AGCAGGGGTGAGAGTCTTTG
IFNB1	AGTAGGCGACACTGTTCGTG	GCCTCCCATTCAATTGCCAC
IFNG	GGGTAACTGACTTGAATGTCC	TTTTCGCTTCCCTGTTTTAG
EREG	TGGACATGAGTCAAAACTACT	GAAGTGTTCACATCGGACACC
IL8	ACTGAGAGTGATTGAGAGTGGAC	AACCCTCTGCACCCAGTTTTC
TGFB1	GGAAATTGAGGGCTTTCGCC	CCGGTAGTGAACCCGTTGAT
TGFB2	AAGCTGAAGCTCACCAGTCC	GCGGGATGGCATTTTCGGAG
TGFB3	ATTCGACATGATCCAGGGGC	CTGGCCGAAGGATCTGGAAG
TGFA	AGGTCCGAAAACACTGTGAGT	AGCAAGCGGTTCTTCCCTTC
IL1B	CCAAACCTCTTCGAGGCACA	GCTGCTTCAGACACTTGAGC
IL1A	GCGTTTGAGTCAGCAAAGAAGTC	GGAGTGGGCCATAGCTTACA
EGF	CTTGGGAGCCTGAGCAGAAA	GCACAAGTGTGACTGGAGGT
HGF	GAATGACACTGATGTTCCTTTGG	GGATACTGAGAATCCCAACGC
FGF1	AAAGCGTGGGGGAGGTGTAT	ATTTGGTGTCTGTGAGCCGT
IL18	CAGATCGCTTCCTCTCGCAA	CCAGGTTTTCATCATCTTCAGCTAT
VEGFA	TAAGTCCTGGAGCGTTCCCT	ACGCGAGTCTGTGTTTTTGC
GAPDH	GGAGCGAGATCCCTCCAAAAT	GGCTGTTGTCATACTTCTCATGG

Supplementary Table 5. Oligonucleotide sequences for ChIP-qPCR analysis.

TNF region	Forward primer (5′-3′)	Reverse primer (5′-3′)
Promoter	AGGGCCCACTACCGCTTC	GTGTGCCAACAACTGCCTTTA
Control	GCCAGGATGTGGAGAGTGAAC	GTTCAGCCACTGGAGCTGC
